# Effects of Endophytic *Bacillus Subtilis* and Salicylic Acid on Postharvest Diseases (*Phytophthora infestans, Fusarium oxysporum*) Development in Stored Potato Tubers

**DOI:** 10.3390/plants9010076

**Published:** 2020-01-07

**Authors:** Oksana Lastochkina, Andrey Baymiev, Aysylu Shayahmetova, Darya Garshina, Igor Koryakov, Irina Shpirnaya, Liudmila Pusenkova, Il’dar Mardanshin, Cemal Kasnak, Recep Palamutoglu

**Affiliations:** 1 Ufa Federal Research Centre of the Russian Academy of Sciences, Bashkir Research Institute of Agriculture, Ufa 450059, Russia; A9610470727@yandex.ru (A.S.); dariya.greatfire@mail.ru (D.G.); l.pusenkova@mail.ru (L.P.); ildar.mardanshin1966@yandex.ru (I.M.); 2 Institute of Biochemistry and Genetics, Ufa Federal Research Centre of the Russian Academy of Sciences, Ufa 450054, Russia; baymiev@anrb.ru (A.B.); koryakov_igor@mail.ru (I.K.); 3 Department of Biology, Bashkir State University, Ufa 450074, Russia; i-shia@yandex.ru; 4 Department of Nutrition and Dietetics, Health Science Faculty, Afyonkarahisar Health Science University, 2078 Afyon, Turkey; ckasnak@gmail.com (C.K.); receppalamutoglu@hotmail.com (R.P.)

**Keywords:** endophytic *Bacillus subtilis*, salicylic acid, postharvest diseases, *Phytophthora infestans*, *Fusarium oxysporum*, resistance, potatoes

## Abstract

Postharvest diseases of potato lead to significant food and economic losses worldwide. The exogenous application of eco-friendly methods plays an important role in the control of postharvest decay. In this work the effects of endophytic bacteria *B. subtilis* (10-4, 26D) were studied in the context of two application parameters: concentration, with a range between 10^3^–10^8^ CFU/mL tested, and synergistic effects of the signal molecule salicylic acid (SA) (0.05 mM) on potato tubers’ resistance to *Phytophthora infestans* and *Fusarium oxysporum* during storage. The experiments were carried out on hydroponically grown potato (*Solanum tuberosum* L.) mini-tubers. This study demonstrates the suppressive effect of *B. subtilis* (10-4, 26D) on diseases of potato during storage and reveals that this effect happens in a dose-dependent manner, both individually and in combination with SA. The most effective concentrations of *B. subtilis* for suppression of both *Ph.* *infestans* and *F. oxysporum* are 10^8^ CFU/mL (10-4 and 26D), 10^7^ CFU/mL (10-4 + SA) and 10^6^ CFU/mL (26D + SA). The ability of *B. subtilis* (10-4, 26D) to effectively penetrate and colonize the internal tubers’ tissues when applied immediately prior to storage, and the ability of SA to accelerate these processes, have been proven. *B. subtilis* (10-4, 26D), individually and in compositions with SA, increased ascorbic acid content and decreased pathogen-induced proline accumulation and lipid peroxidation in tubers. This indicates a protective effect conferred to cells against reactive oxygen and an extension of aging processes, manifested by a prolonged shelf life and extended preservation of fresh appearance.

## 1. Introduction

Potato (*Solanum tuberosum* L.) is one of the most valuable crops (after wheat, rice, and maize) with great importance in ensuring food security worldwide [1,2]. Potatoes are an excellent source of nutrients and vitamins, but their year-round availability depends on storage at an industrial scale, especially in countries that depend on annual crops. About half of all harvested tubers are stored for up to 11 months [3]. Losses (up to 50–60%) of potatoes from postharvest diseases can occur at any time during storage, from harvesting to consumption, and are one of the most acute problems of modern agriculture and the food industry on the whole planet [4]. Most of the losses that occur during storage are due to infestation by such harmful phytopathogenic fungi as *Phytophthora infestans* (causal agent of late blight) [5,6] and *Fusarium oxysporum* (causal agent of fusarium wilt and dry rot) [7]. *Ph. infestans* is considered to be the most significant potato pathogen worldwide [4] and was responsible for the Great Potato Famine of the late 1840s [8]. Losses associated with *Fusarium*-caused diseases range from 6% to 25%, and sometimes reach up to 60% during long-term storage [7]. Besides the damage inflicted on tubers, *Fusarium* also produces mycotoxins that are harmful to humans and animals [9,10]. Traditionally applied chemical fungicides to reduce disease development in stored food products are, in many cases, hazardous to humans, animals and the environment [11]. Due to their toxicological risk, chemicals registered for postharvest use are severely limited, and in some European countries completely prohibited altogether [12]. It follows that the use of environmentally friendly and safe approaches to induce natural defense mechanisms of the plant organisms play an important role in disease control [13].

The beneficial microorganisms *Bacillus* spp. are highly efficacious, science-based and research-led alternative to synthetic fungicides for biological control of postharvest diseases. Among the members of the *Bacillus* genus, *B. subtilis* strains have special potential to be bio-active and eco-friendly agents for controlling postharvest decays due to (i) their ability to induce host-plants’ natural defense response mechanisms to a wide range of pathogens [14,15,16,17,18,19] and abiotic (drought, salinity, extreme temperatures, toxic metals, etc.) stresses [15,20,21,22,23,24,25,26]; (ii) their generally recognized status as a safe microorganisms to use in the food industry [13,27,28,29]. *B. subtilis* has been shown to increase the resistance of a wide range of fresh-cut stored fruits/vegetables to various diseases and abiotic stresses during handling, transportation, and storage, with the effect of protecting stored food from postharvest decays and prolonging its shelf-life [13,27,29,30,31]. For example, the ability of *B. subtilis* to suppress the development of *Botrytis cinerea* and *B. mali*-causing grey mold has been demonstrated in strawberry, pear, apple, and tomato [32,33,34,35,36,37,38]. In addition to the protective effects of *B. subtilis* against postharvest disease, temperature fluctuations, and mechanical injury associated with transportation, unloading, packaging, and storage, some studies suggest *B. subtilis* carries additional intrinsic potential to increase vegetable/fruit sets [27,39]. The biocontrol and beneficial effects of *B. subtilis* strains can be attributed to their ability to occupy the same niche as many pathogens, their capacity to produce a wide range of bio-active substances with antibiotic activities including siderophores, lipopeptides, enzymes, 1-aminocyclopropane-1-carboxylate (ACC) deaminase, and exopolysaccharides, their regulation of phytohormone biosynthesis pathways and modulation of ethylene levels in plant organisms, and their influence on the emission of volatile organic compounds [16,18,25,27,29,40,41,42,43]. These substances induce various physiological features in host plant metabolism without causing adverse effects on the environment and human health [13,44]. Furthermore, *B. subtilis* produces endospores that are resistant to dynamic physical and chemical treatments, such as heat, desiccation, organic solvents, and UV irradiation, and therefore maintain their ability to trigger defense responses in host plants, even under unfavorable conditions [40,45,46]. This makes it possible to easily formulate and store *Bacillus*-based biological products in which the potency of the bio-active component against pathogens is preserved. The most effective agents in the biocontrol of postharvest diseases can be endophytic *B. subtilis* living inside plant tissues, which allows them to be less dependent on external environmental factors (compared with rhizospheric and phyllospheric strains) while simultaneously exhibiting economically “useful” properties [43,47] without adversely affecting the host plant, the environment and human health [13,44,48]. Endophytic bacteria colonize the same ecological niches as phytopathogenic microorganisms and being in the endosphere, have a significant advantage over epiphytic organisms living in the rhizosphere and phyllosphere due to the stable pH, humidity, flow of nutrients, and the lack of competition from a large number of other microorganisms. Moreover, bacterial endophytes, once established in the plant tissue, may contribute to the formation of long-term protection against adverse environmental factors over time [49,50,51,52]; therefore, they are considered as promising agents for biocontrol of phytopathogens [13]. Commercial products based on *B. subtilis* for postharvest application are already on the market [13]. However, their role in controlling postharvest diseases and the underlying mechanisms regulating fruit/vegetable storage quality remains largely unknown. Meanwhile, one of the main factors hampering the development of products based on *B. subtilis* is the lack of data from a systematic study of the mechanisms underlying the relationships the govern the “endophytic bacteria *B. subtilis*–host–plant–phytopathogen” system. Moreover, due to the fact that it is difficult to choose an individual effective microbial strain with a wide spectrum of activity against a number of pathogens, it is also of interest to combine *B. subtilis* with other biological methods. Of particular interest is the use of *B. subtilis* together with signal molecules with pronounced anti-stress activity, such as salicylic acid (SA) [13,53]. Despite the fact that there is information in the literature on the effectiveness of using SA in increasing the consumer properties and resistance of various types of vegetables, fruits, and berries to diseases and stresses during storage [54,55,56], practically no data exist about the effect of endophytic *B. subtilis* together with SA on stored potatoes’ postharvest physiology and resistance to disease.

The aim of this work was to study the influence of endophytic bacteria *B. subtilis* strains 10-4 and 26D, both individually and in combination with the natural signal molecule SA, on the development of the most economically harmful postharvest diseases of potato caused by *Ph. infestans* (late blight) and *F. oxysporum* (fusarium wilt and dry rot) during storage.

## 2. Results and Discussion

### 2.1. Effect of Endophytic B. subtilis (Strains 10-4, 26D) at Different Concentrations, Individually and in Compositions with SA, on the Development of Ph. infestans and F. oxysporum in Potato Tubers during Storage

**Figure 1:**
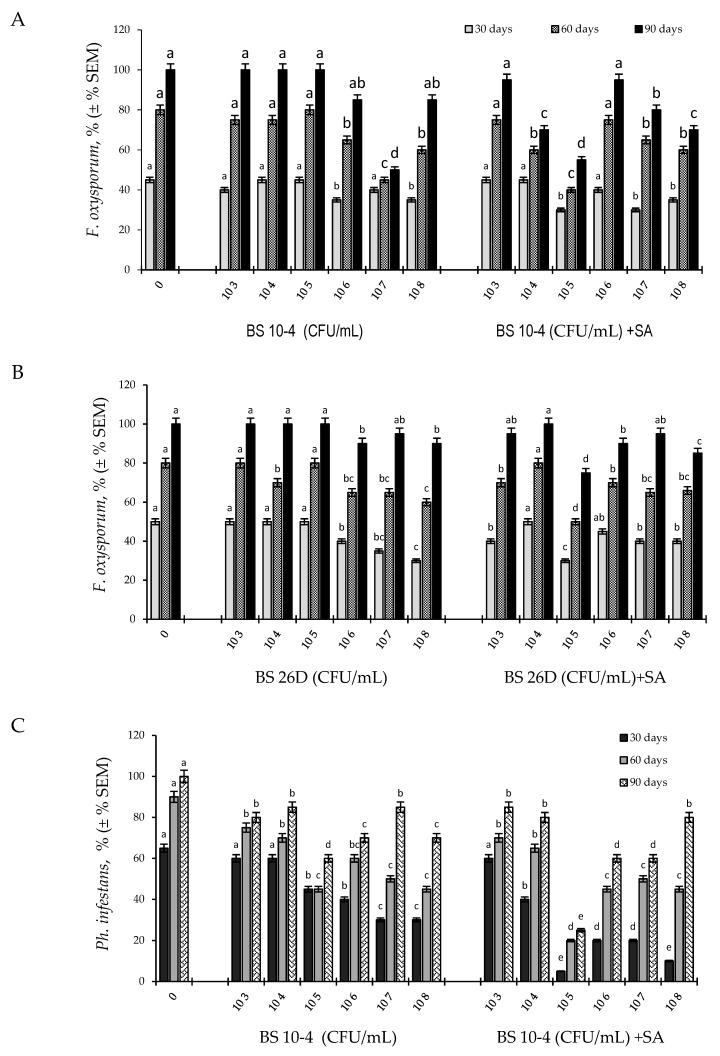

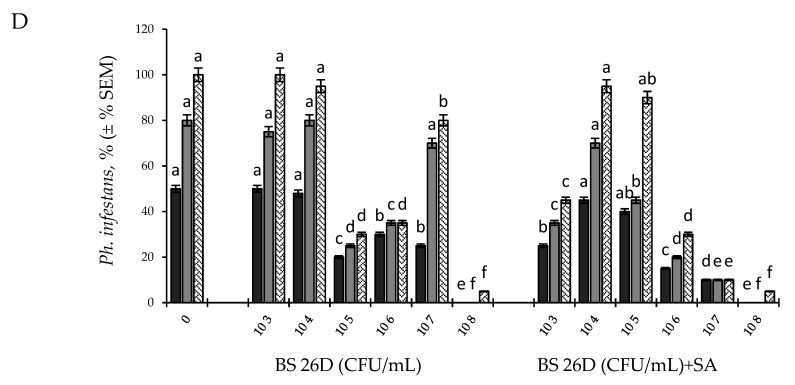
The effect of *B. subtilis* 10-4 (BS 10-4) (**A**,**C**) and *B. subtilis* 26D (BS 26D) (**B**,**D**) at different concentrations (0, 10^3^, 10^4^, 10^5^, 10^6^, 10^7^, and 10^8^ CFU/mL), individually and in compositions with salicylic acid (SA) (0.05 mM,) on the development of *F. oxysporum* (**A**,**B**) and *Ph. infestans* (**C**,**D**) in potato tubers during storage (time of storage 3 months). Bars indicate mean values of three repetitions ± SEM. Different letters indicate a significant difference between the means at the probability level of *p*  <  0.05.

This study analyzed of the effect of endophytic *B. subtilis* (strains 10-4 and 26D) at a wide range of concentrations, both individually and in their compositions with SA, on *F. oxysporum* (Figure 1A,B) and *Ph. infestans* development in potatoes during the storage period. The results revealed the dose-dependent nature of *B. subtilis* (10-4, 26D) activity, separately and in compositions with SA (Figure 1). It was discovered that the most effective concentrations for the suppression of both *Ph. infestans* and *F. oxysporum* development are 10^8^ CFU/mL (for *B. subtilis* 10-4 and *B. subtilis* 26D), 10^7^ CFU/mL (for *B. subtilis* 10-4 + SA) and 10^6^ CFU/mL (for *B. subtilis* 26D + SA).

Infection of tubers with phytopathogenic fungi *Ph. infestans* and *F. oxysporum* resulted in typical disease symptom development. These symptoms manifest first as brown blurry spots from the skin deep into the tuber (late blight) and gray (brown) putrefactive depressed spots with a white coating, under which the flesh becomes dry and rotten. Later, the skin wrinkles and white spores of the fungus appear on it, which can dissipate and infect neighboring tubers (fusarium). This becomes clearly visible after 3–6 months storage at a temperature of +4 °C at which point tubers are completely infected. So, tuber infection with *Ph. infestans* and *F. oxysporum* over time led to a gradual increase in lesion symptomatic of late blight and fusarium dry rot which reached 100% by 3–6 months of storage (Figure 1). Bacterization with *B. subtilis* 10-4 and 26D reduced the intensity of late blight and fusarium disease development, manifested as a decrease in lesion area or even in their absence; however, not in all tested concentrations (Figure 1A,B). The degree of both late blight and fusarium disease suppression by *B. subtilis* 10-4 and *B. subtilis* 26D depended on the concentrations tested, on the presence of SA, and the type of affected phytopathogens. It was discovered that application of *B. subtilis* strain 10-4 and strain 26D in concentrations of 10^3^, 10^4^, and 10^5^ CFU/mL had practically no effect on tubers infected with *F. oxysporum* and did not prevent the development of the disease symptoms (Figure 1A,B).

Whereas, starting from a concentration of 10^6^ CFU/mL and above, there was a visible decrease in the manifestation of the visual symptoms of this disease by 15–25% (strain 26D) and 20–50% (strain 10-4) in comparison with the control. Moreover, the most effective treatment against fusarium infection was strain 10-4 (10^7^ CFU/mL), which inhibited the development by 50%, compared to the 25% inhibition conferred by strain 26D at 10^6^ and 10^7^ CFU/mL concentrations (Figure 1A,B).

With regards to the *Ph. infestans* (late blight) infected variants, the picture was different (Figure 1C,D). Strain 10-4 reduced the degree of *Ph. infestans* development up to 20–50% in all tested concentrations (10^5^ CFU/mL had the best effect (reduction of the disease by 50%) and 10^3^, 10^6^, and 10^8^ CFU/mL (decrease by about 30%), and strain 26D showed a visible protective effect in the form of a 10–95% reduction in the development of the disease starting from a concentration of 10^4^ CFU/mL (best effect 10^8^ CFU/mL (reduction of the disease by 95%)) and 10^5^, 10^6^ CFU/mL (decrease by 65–70%)) (Figure 1C,D). At the same time, 10^8^ CFU/mL was determined to be the most optimal dosage for *B. subtilis* strains 10-4 and 26D, capable of reducing the development of both diseases (late blight and fusariosis) (Figure 1). According to literature data in most harvested fruits and vegetables, the bio-control activity of bacterial strains is elevated by increasing their concentration as well as the reduced level of pathogens. The most effective concentration in controlling postharvest fruit/vegetable diseases is generally considered to be 10^7^–10^8^ CFU/mL [57]. The effectiveness of antagonists mainly depends on their ability to outperform pathogens based on their capacity for rapid growth and survival under unfavorable conditions and is strongly dependent on their initial concentration when applied to the wound site [58].

**Figure 2:**
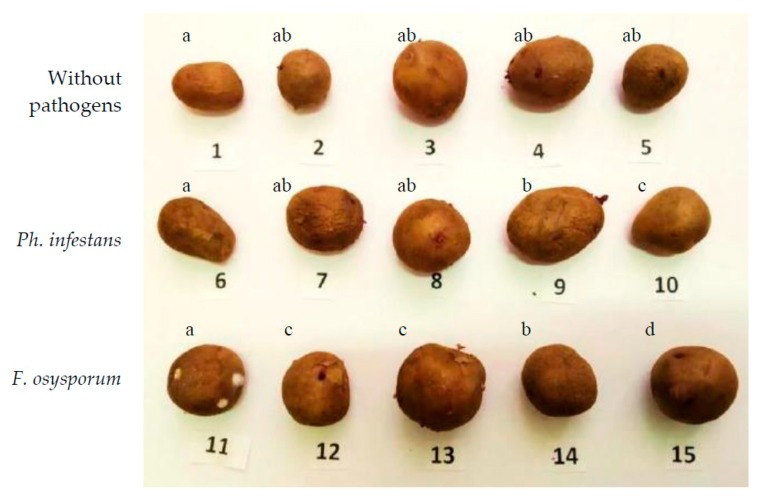
The effect of selected concentrations of *B. subtilis* 10-4 (10^8^ CFU/mL) and 26D (10^8^ CFU/mL) separately and compositions with SA (*B. subtilis* 10-4 (10^7^ CFU/mL) + SA; *B. subtilis* 26D (10^6^ CFU/mL) + SA) on the development of *F. oxysporum* and *Ph. infestans* in potato tubers during storage (after 5 months storage). **1**—Control (without treatments); **2**—strain 26D; **3**—strain 10-4; **4**—strain 26D + SA; **5**—strain 10-4 + SA; **6**—*Ph. infestans*; **7**—*Ph. infestans*+strain 26D; **8**—*Ph. infestans*+strain 10-4; **9**—*Ph. infestans*+strain 26D + SA, **10**—*Ph. infestans*+strain 10-4 + SA; **11**—*F. oxysporum*; **12**—*F. oxysporum*+strain 26D; **13**—*F. oxysporum*+strain 10-4; **14**—*F. oxysporum*+strain 26D + SA; **15**—*F. oxysporum*+strain 10-4 + SA. In figure presented the pictures of medium tubers from five repetitions ± SEM. Different letters indicate a significant difference between the means at the probability level of *p * <  0.05.

**Figure 3:**
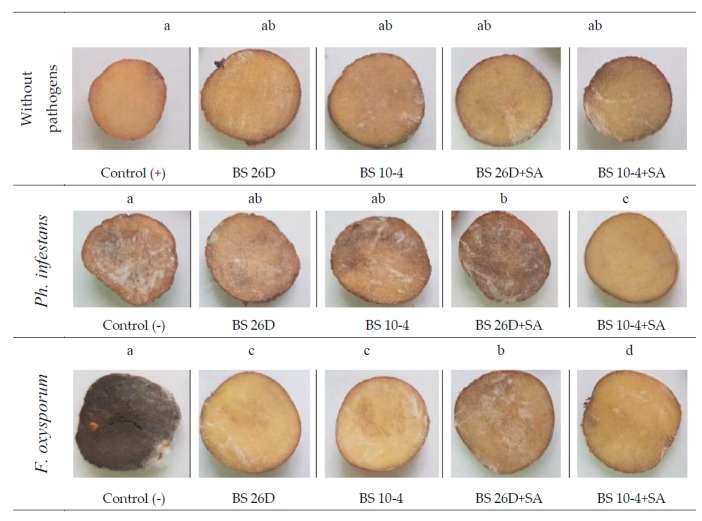
The effect of selected concentrations of *B. subtilis* 10-4 (10^8^ CFU/mL) and 26D (10^8^ CFU/mL), separately and compositions with SA, (*B. subtilis* 10-4 (10^7^ CFU/mL) + SA; *B. subtilis* 26D (10^6^ CFU/mL) + SA) on the development of *F. oxysporum* and *Ph. infestans* in potato tubers during storage (after 5 months storage). In figure presented the pictures of medium tubers from five repetitions ±  SEM. Different letters indicate a significant difference between the means at the probability level of *p*  <  0.05.

When *B. subtilis* was used together with SA, bacilli apparently began to suppress the development of *F. oxysporum* at all concentrations with most visible effect in treatments using 10^5^ CFU/mL for both strains 10-4 and 26D. Particularly, *F. oxysporum* development was suppressed by *B. subtilis* 10-4 (10^5^ CFU/mL) and 26D (10^5^ CFU/mL) up to 50% and 25%, respectively (Figure 1A,B). A parallel outcome was observed from the combined application of *B. subtilis* strain 10-4 with SA against *Ph. infestans* development in stored tubers (Figure 1C). Contrary, combined application of strain 26D with SA, most effectively suppressed *Ph. infestans* development at a concentration of *B. subtilis* 10^8^ CFU/mL (Figure 1D). Thus, the results of this study support the combined use of *B. subtilis* with SA at a concentration of 10^5^ CFU/mL (for strain 10-4, suppression of fusarium and late blight up to 50% and 70%, respectively) and 10^5^ CFU/mL (for strain 26D, suppression of fusarium by 25%), as well as 10^6^–10^8^ CFU/mL (for strain 26D, suppression of late blight by 70–95%). Moreover, the most optimal treatments in suppressing both late blight and fusariosis were combinations of SA+strain 10-4 (10^7^ CFU/mL) and SA+strain 26D (10^6^ CFU/mL). These findings indicated that in combination with SA, the protective effects of *B. subtilis* begin to appear at lower concentrations. This is probably due to the ability of SA to trigger additional protective reactions or enhance the action of bacterial strains in inhibiting the development of diseases, while the mechanisms of these strains are probably different and may be associated with the nature of the synthesized compounds and the pathways involved in the implementation of these protective effects. However, it is interesting that by the 5-6th month of storage following *Ph. infestans* infection, tubers treated with *B. subtilis* 10-4 + SA looked completely healthy and fresh, while in variants with *B. subtilis* 26D + SA treatment, the picture was different, and traces of damage were visible on the tubers (Figure 2). It can be assumed that the mechanisms of action differ between strains 10-4 and 26D and that in the compositions with SA, the protective effect against late blight is enhanced in one case (strain 10-4 + SA), and not the other (strain 26D + SA) (Figure 3). This finding allows for the possibility that this effect may be associated with the ability of the strains to produce their own phytohormones [13]. It is likely that if strain 10-4 produces SA, then the addition of exogenous SA can enhance its protective effect; if strain 26D produces jasmonic acid (JA), the addition of SA to the composition may have the opposite effect, since JA and SA are antagonists. This assumption, of course, requires further detailed study and confirmation.

In our experiments the freshest and most healthy-looking tubers (even after 5–6 months of storage and under *Ph. infestans* and *F. oxysporum* -infected conditions) (Figure 2) were observed in combined *B. subtilis* 10-4 and SA treatments.

It can be assumed that SA has preservative properties or, that by being a potential inhibitor of biosynthesis and the action of ethylene; it slows down the maturation and aging processes, thereby improving the quality of products [56]. This is supported by data on the ability of SA to increase the resistance of various types of vegetables, fruits, and berries to diseases and stresses during storage as measured by degree of preservation or even increases in nutritional value [21,54,55,56]. For example, Dokhanieh et al. [59] showed that SA-treated serdolin cherry is characterized by a significantly higher content of total phenols, flavonoids, anthocyanins, ascorbic acid, etc. Apparently, the reported ability of *B. subtilis* 10-4 and 26D to more effectively suppress the development of late blight and fusariosis in stored tubers when these bacteria were used together with SA, may be due to the fact that SA, as a natural and safe signal molecule, enhances and accelerates the spread of the systemic immunizing effect of *B. subtilis* in potato tuber tissues. In addition, it is possible that SA can activate additional protective mechanisms responsible for inhibiting the aging process and prolonging the life of products while maintaining freshness. This, however, requires further detailed study.

Due to the fact that as the most optimal treatments for the simultaneous suppression of late blight and fusarium were determined to be 10^8^ CFU/mL (for strains 10-4 and 26D), 10^7^ CFU/mL (for strain 10-4 + SA) and 10^6^ CFU/mL (for strain 26D + SA) further microbiological, molecular, and physiological-biochemical studies would continue to use these established concentrations.

### 2.2. The Ability of B. subtilis (10-4 and 26D) to Colonize the Internal Tissues of Potato Tubers Treated before Storage with B. subtilis and B. subtilis + SA under Normal and Pathogens (Ph. infestans and F. oxysporum)-Infected Stored Conditions

**Figure 4:**
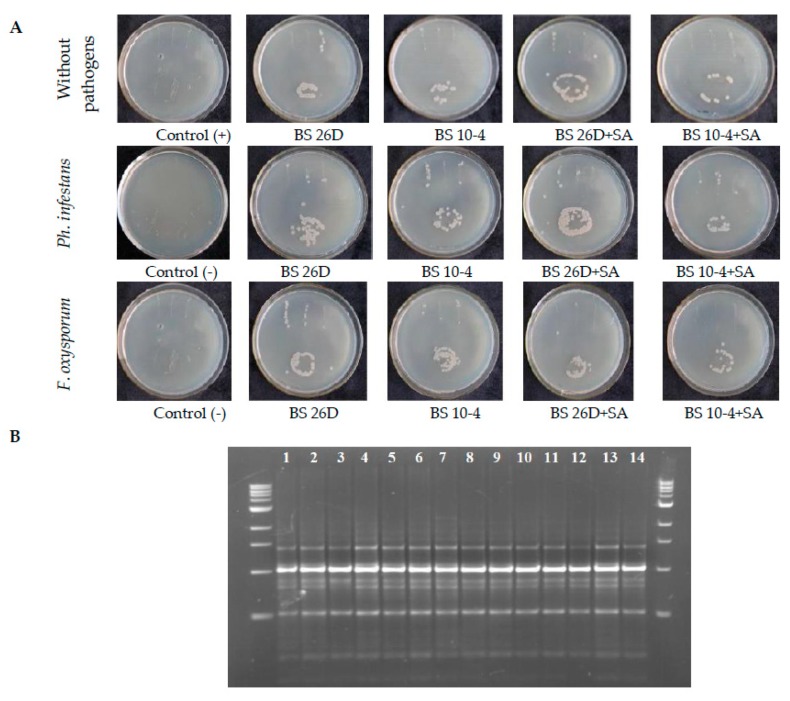
Assessment of the ability of *B. subtilis* 10-4 (BS 10-4) and 26D (BS 26D) to colonize the internal tissues of potato tubers treated before storing with BS 10-4, 26D and SA under normal and pathogens (*Ph. infestans* and *F. oxysporum*)-infected conditions. **A**—bacterial growth at the site of imprints of slices of surface-sterilized tubers inoculated with BS 10-4 and 26D before storage (control—the absence of bacterial growth at the site of imprints of sections of surface-sterilized tubers not inoculated with *B. subtilis* before storing); **B**—electrophoregram of PAAG after RAPD-PCR analysis: **1**—DNA of the original BS 10-4; **2**—DNA of the original BS 26D; **3**—DNA of the corresponding bacterial strains isolated from the prints of tubers treated with BS 10-4; **4**—DNA of the corresponding bacterial strain isolated from the prints of tubers treated with BS 26D; **5**—BS 10-4 + SA; **6**—BS 26D + SA; **7**—*Ph. infestans*+BS 10-4; **8**—*Ph. infestans*+BS 26D; **9**—*Ph. infestans*+BS 10-4 + SA; **10**—*Ph. infestans*+BS 26D + SA; **11**—*F. oxysporum* + BS 10-4; **12**—*F. oxysporum* + BS 26D; **13**—*F. oxysporum*+BS 10-4 + SA; **14**—*F. oxysporum* + BS 26D + SA. In figure presented the pictures from three repetitions ± SEM.

Colonization of the internal tissues of plants by bacteria is one of the most important indicators of their endophytic properties and a factor influencing biological activity in plant-microbial relationships [25,43]. Using the prints of slices of surface sterilized tubers and quantitative accounting (titer *B. subtilis*), it was experimentally shown that *B. subtilis* 10-4 and 26D, when applied immediately prior to storage, effectively penetrate the internal tissues of the tubers and colonize them from the inside (Figure 4A).

This data clearly demonstrates that the combined use of strains with SA helps significantly increases their ability to penetrate and colonize internal tissues, which is especially noticeable (already at 2 weeks of storage) in the variant strain 26D + SA and especially in *Ph. infestans* infected tubers. It is possible that one of the reasons for enhanced efficiency of *B. subtilis* when used with SA, against the development of late blight and fusariosis in tubers during storage, is the ability of SA to accelerate the penetration and colonization of internal tissues by *B. subtilis*, leading to significantly earlier competition with pathogenic microorganisms. It was observed in both *Ph. infestans* and *F. oxysporum* treatments that the rate and nature of penetration/colonization of the same *B. subtilis* strains differed in “healthy” and infected tubers. After 2 weeks of storage, *B. subtilis,* in almost all tested configurations, concentrated to a greater extent closer to the surface of the tubers (Figure 4A) (this is possibly due to the fact that there was competition with pathogenic microorganisms for space and only after their displacement by the bacilli continued to further populate the tissues), in some cases, the path along which penetration to the center and colonization was clearly visible. An assessment of colonizing ability after 3 months of storage revealed that over time, bacteria actually continued to penetrate further and colonize tissues more intensively-throughout the entire inner part in some cases. A significant increase in number was visually apparent over this period. It was revealed that SA accelerates of the process of colonization by bacteria *B. subtilis* 10-4 and 26D as evidenced by an increase in their titer during storage. This phenomenon was observed in the internal tissues of both “healthy” and infected (*Ph. infestans* and *F. oxysporum*) potato tubers, with the maximum effect observed in variants strain 26D + SA (and especially in conditions of infection with *Ph. infestans*).

As evidence that bacteria isolated from the internal tissues of tubers were progeny of the initial *B. subtilis* 10-4 and 26D strains this study used RAPD-PCR analysis to confirm that the bacteria isolated from the internal tissues of tubers, and the experimentally applied 10-4 and 26D strains of *B. subtilis,* had the same molecular profile (Figure 4B). It was found that tubers not treated with bacteria (control, SA, *Ph. infestans*, and *F. oxysporum*) did not contain endophytic *B. subtilis*. Accordingly, in the used hydroponically grown mini-tubers, there were no native endophytic bacilli. These findings indicate that in the early stages of colonization by *Ph. infestans* and *F. oxysporum*, the bacteria *B. subtilis* (strains 10-4 and 26D) (especially in mix with SA) conduct an effective competition for nutrients and space from the inside, suppressing their development. This hypothesis is supported by in vitro studies demonstrating that bacterial inoculants take up nutrients faster than pathogens, leading to the inhibition of germination of pathogen spores at the wound site [60,61]. Moreover, a fundamental strategy for nutrient competition is the attachment of microbial antagonists to the hyphae of a pathogen due to the fact that the antagonists feed on nutrients faster than the target pathogen, thus hampering spore germination and pathogen growth [60,62]. Nevertheless, in certain cases such as *Aureobasidium pullulans* against *Botrytis cinerea*, *Rhizopus stolonifer*, *Penicillium expansum*, and *Aspergillus niger*, which infect table grapes and *P. expansum* and *B. cinerea* on apple fruit, direct physical interaction is not required for the antagonistic activity [63]. In such circumstance’s antagonism does not occur via direct attachment of antagonistic microorganism to pathogen hyphae. Rather, it is highly likely that other alternative mechanisms, such as the production of a wide range of biologically active molecules, including antibiotics, biosurfactants, siderophores, hydrogen cyanide, and hydrolases increase their advantage against pathogens as they compete for a suitable niche for colonization [63,64].

### 2.3. Change in Count of B. subtilis in Healthy and Infected (by Ph. infestans and F. oxysporum) Tubers over Storage Time and under Exogenous SA Influence

**Figure 5:**
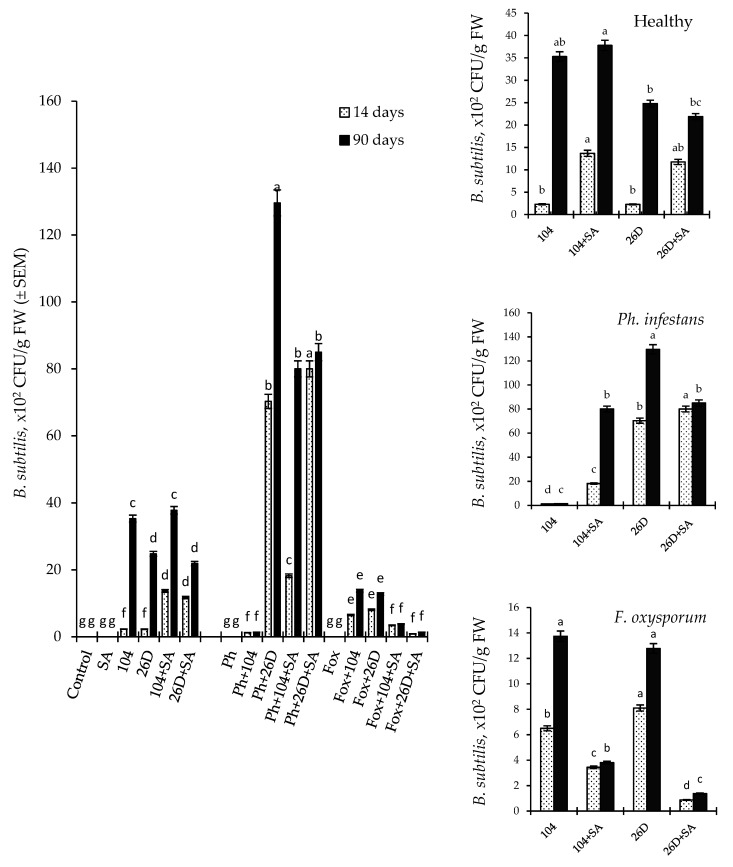
Change in the titer of endophytic *B. subtilis* bacteria (strain 10-4 and 26D) in stored healthy and infected with *Ph. infestans* (Ph) and *F. oxysporum* (Fox) potato tubers treated immediately before storage with *B. subtilis* 10-4 (104), *B. subtilis* 26D (26D) and their compositions with SA. The bars are the means of three repetitions ± SEM. Different letters indicate a significant difference between the means at the probability level of *p*  <  0.05.

It was found that tubers not treated with bacilli (variants: Control, SA, *Ph. infestans* and *F. oxysporum*) did not contain endophytic *B. subtilis* (Figure 5), which is consistent with the data obtained above by visual assessment of the ability of the studied strains to colonize the internal tissues of tubers using tuber prints (Figure 4). At the same time, in healthy tubers treated before storage with *B. subtilis* 10-4 and 26D, the titer of the bacilli was 2.30 × 10^2^ CFU/g and 2.28 × 10^2^ CFU/g FW, respectively (after 2 weeks of storage) and increased to 35.3 × 10^2^ CFU/g (strain 10-4) and 24.8 × 10^2^ CFU/g (strain 26D) by the third month of storage (Figure 5).

The titer of bacilli also differed depending on the pathogen that affected the tuber. So, under *Ph. infestans*-infected conditions the titer of strain 10-4 was comparable to that in healthy tubers; over time, it changed insignificantly and ranged around 1.22–1.36 × 10^2^ CFU/g. The titer of strain 26D, in contrast, increased over the same time period, going from 70.3 × 10^2^ CFU/g (after 2 weeks of storage), to 129.61 × 10^2^ CFU/g (after 3 months of storage). Under the conditions of infection of tubers with *F. oxysporum*, a similar significant increase in the titer of bacilli did not occur. By the second week of storage in *F. oxysporum*-infected tubers, the titer of bacilli was 6.5 × 10^2^ CFU/g (for strain 10-4), 8.1 × 10^2^ CFU/g (for strain 26D) and by the third month of storage the bacilli titer increased to 13.74 × 10^2^ CFU/g (for strain 10-4) and 12.78 × 10^2^ CFU/g (for strain 26D). It was found that the use of SA promotes a significant (up to 130-fold) increase in the titer of strains 10-4 and 26D, both in healthy and *Ph. infestans*-infected tubers (Figure 5) in comparison with variants where only bacteria were used. Thus, the titer of bacilli in healthy tubers for the variant strain 10-4 + SA was 13.69 × 10^2^ CFU/g (2 weeks of storage) and 37.8 × 10^2^ CFU/g (3 months of storage), for the composition strain 26D + SA was 11.76 × 10^2^ CFU/g (2 weeks of storage) and 21.9 × 10^2^ CFU/g (3 months storage). In tubers infected by *Ph. infestans* the titer of bacilli in the variant strain 10-4 + SA was 18.23 × 10^2^ CFU/g (2 weeks of storage) and 80.0 × 10^2^ CFU/g (3 months of storage), in the variant strain 26D + SA was 80.0 × 10^2^ CFU/g (2 weeks of storage) and 85.0 × 10^2^ CFU/g (3 months of storage). In contrast, tubers infected by *F. oxysporum* exhibited no such increase, even decreasing in some cases. So, by the third month of storage, the *B. subtilis* titer in late blight-infected tubers in the variant strain 10-4 + SA amounted to 3.44 × 10^2^ CFU/g (2 weeks storage) and 3.81 × 10^2^ CFU/g (3 months of storage), in the variant strain 26D + SA was 0.87 × 10^2^ CFU/g (2 weeks of storage) and 1.38 × 10^2^ CFU/g (3 months of storage) and quantitatively the values were even lower than in healthy control stored tubers (Figure 5).

Thus, the data obtained from this study indicate that *B. subtilis* bacterial strains 10-4 and 26D successfully colonize the internal tissues of tubers. However, the nature of their penetration/colonization (titer change) depends both on the characteristics of the strain itself, the presence of the signaling molecule SA, the type of pathogen that affects the tuber, and, possibly, varietal characteristics of the plant organism itself (resistance, susceptibility to pathogens, etc.). One of the mechanisms of protection by *B. subtilis* bacterial strains 10-4 and 26D, both individually and in compositions with SA from the causative agents of late blight and fusariosis during storage, is their ability in the early stages of pathogen damage to compete effectively for nutrition and space with pathogenic fungi internally, which in some cases almost completely suppresses their development.

Moreover, as was mentioned above, endophytes have a significant advantage over epiphytic (surface) organisms living in the rhizosphere and phyllosphere due to the stable pH, humidity, nutrients and the lack of competition from a large number of microorganisms. It is believed that endophytic bacteria, once embedded in plant tissue, establish their long-term protection against environmental factors over a period of time, which, in particular, can be manifested in better preservation of fruits and vegetables during storage [13,21,50,52]. So, the observed protective effect of the studied endophytic *B. subtilis* (strains 10-4 and 26D) and SA on potato tubers during storage against late blight and fusariosis for a long time may be associated both with their ability to penetrate into internal tissues and with their internal regulation of physiological processes responsible for inducing defense mechanisms against pathogens.

### 2.4. Effect of B. subtilis (10-4 and 26D), Individually and in Compositions with SA, on Lipid Peroxidation (MDA) in Stored Healthy and Pathogens (Ph. infestans and F. oxysporum)-Infected Potato Tubers

**Figure 6:**
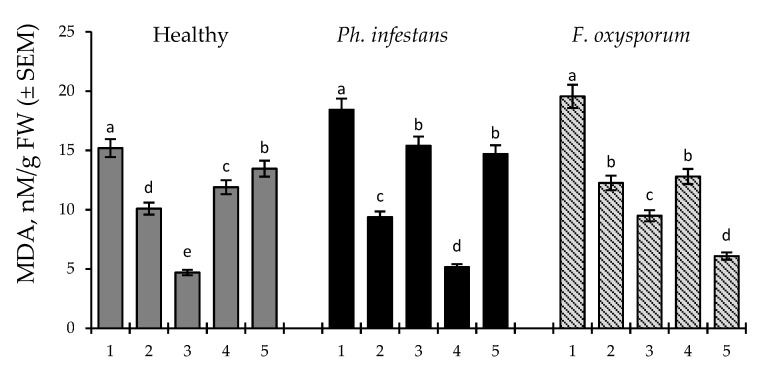
Effect of endophytic *B. subtilis* 10-4 and 26D, both individually and in compositions with SA, on MDA content in healthy and pathogens (*Ph. infestans* and *F. oxysporum*)-infected stored potato tubers (time of storage 5 months). **1**—control (non-inoculated with *B. subtilis*), **2**—*B. subtilis* 10-4; **3**—*B. subtilis* 10-4 + SA; **4**—*B. subtilis* 26D; **5**—*B. subtilis* 26D + SA. The bars are the means of three repetitions ± SEM. Different letters indicate a significant difference between the means at the probability level of *p*  <  0.05.

As shown in Figure 6, the tubers infected with phytopathogenic fungi (*Ph. infestans*, *F. oxysporum*) exhibited an increased level of malondialdehyde (MDA), a product of membrane lipid peroxidation. This was quite expected due to two know an phenomenon (i) the environmental and physical changes before and after harvest induced the production of reactive oxygen species (ROS), which in turn cause oxidative damage during the postharvest stages of immature fruits and vegetables, inducing decay of the product and loss of quality; (ii) the formation of ROS (hydrogen peroxide (H_2_O_2_), superoxide radicals, etc.), one of the earliest responses of plant cells to pathogens infection, which induce range of defense responses in plants, including the synthesis of a whole spectrum of protective compounds [13,19,39,44,45,47]. If ROS production increases dramatically, as occurs under environmental stresses, hydroxyl radical reacts with membrane lipids, inducing their peroxidation—the final product of which is MDA [13,25,53]. Thus, the development of the protective reactions of a plant organism to pathogens can be assayed by the degree of MDA accumulation.

It was revealed that application of *B. subtilis* 10-4 and 26D, both individually and in compositions with SA, helped to reduce pathogen-induced increase of MDA in healthy and infected stored tubers (Figure 6). The findings indicated a reduction of oxidative stress in stored tubers treated before storage with *B. subtilis* 10-4, *B. subtilis* 10-4 + SA, *B. subtilis* 26D and *B. subtilis* 26D + SA. Perhaps the protective effect of these bacteria, both individually and in compositions with SA, may be related to the modulation activity of oxidative enzymes under their influence, so they can control the level of H_2_O_2_, which induces lipid peroxidation.

### 2.5. Effect of B. subtilis (10-4 and 26D) and B. subtilis + SA on Proline (Pro) Content in Healthy and Pathogens (Ph. infestans and F. oxysporum)-Infected Stored Tubers

An important biochemical marker of plant disease resistance is the accumulation of Pro, a multifunctional stress metabolite that acts as an osmolyte, antioxidant and low molecular weight chaperone involved in maintaining the native structure of enzymes [25,53]. Many studies have reported an increase in Pro content in plant organisms in response to stresses of various natures, and its significance as a factor important to plant survival under extreme situations [13,25,53]. However, data on changes in the Pro content in potato tubers during storage and under conditions of infection with pathogens of late blight and fusariosis and the use of endophytic bacteria *B. subtilis* and *B. subtilis* + SA have not been found in the available literature.

**Figure 7:**
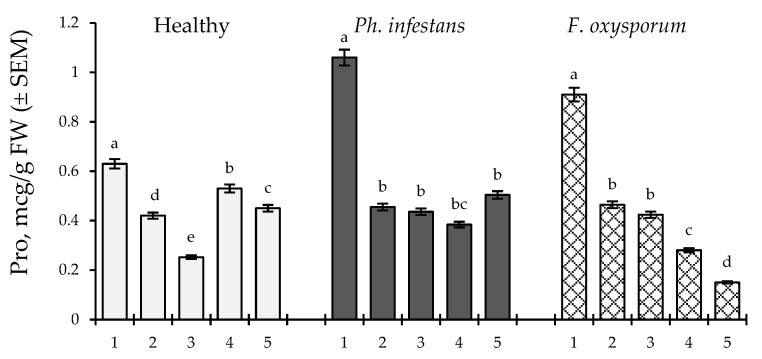
Effect of endophytic *B. subtilis* 10-4 and *B. subtilis* 26D, both individually and in compositions with SA, on Pro content in healthy and infected by *Ph. infestans* and *F. oxysporum* stored potato tubers (time of storage 5 months). **1**—control (non-inoculated with *B. subtilis*), **2**—*B. subtilis* 10-4; **3**—*B. subtilis* 10-4 + SA; **4**—*B. subtilis* 26D; **5**—*B. subtilis* 26D + SA. The bars are the means of three repetitions ± SEM. Different letters indicate a significant difference between the means at the probability level of *p*  <  0.05.

Our results showed that infection of potato tubers by *Ph. infestans* and *F. oxysporum* corresponded with a significant increase in endogenous Pro content during storage (Figure 7). At the same time, tubers treated with *B. subtilis* 10-4 and 26D, both individually and in compositions with SA, exhibited a reduced level of pathogen (*Ph. infestans* and *F. oxysporum*)-induced Pro accumulation. Under the influence of *Bacilli* in healthy tubers (non-infected with pathogens), a slight decrease in the amount of Pro was also observed (Figure 7), which may indicate an important role of this compound in the formation of induced resistance to the causative agents of late blight and fusariosis. Changes in Pro content induced by *B. subtilis* (10-4 and 26D) and *B. subtilis* (10-4 and 26D) + SA along with the role in osmoregulation may also protect the structure of different biomolecules and membranes or act as free-radical scavengers that protect DNA from damaging effects of ROS [53] that, probably, makes a contribution to pathogen suppression in stored tubers, thereby slowing down their aging process.

### 2.6. Effect of Endophytic B. subtilis (10-4, 26D), and Their Compositions with SA, on Ascorbic Acid (AA) Content in Stored Potato Tubers

The non-enzymatic antioxidant AA (vitamin C) can also serve as a biomarker of the physiological state of tubers during storage. In addition, potatoes, vegetables and fruits are its main source for the human body. However, AA is a very labile substance, easily and irreversibly oxidized under the influence of active stressful effects [65]. In this regard, under changing environmental conditions, in a state of reduced functional activity of the plant organism at a low storage temperature, AA level can characterize the response of plant tissues to metabolic products of the studied strains of the antagonist bacteria *B. subtilis*. The only data available in the literature that associates AA content with the ability of *B. subtilis* to increase resistance to damage by pathogens, preventing fruit from withering during storage while maintaining a high level of their consumer properties (in comparison with the control), was demonstrated in a study with lychee fruit in which treatment with *B. subtilis* bacterial cells did not alter the taste of the fruit [27]. A 1.3-fold increase in AA level associated with *B. subtilis* application was observed in potatoes during storage [65]. So, by the time the work began, there was only limited information in the literature about the effect of *B. subtilis* on the AA content, and in combination with SA it was not found at all.

**Figure 8:**
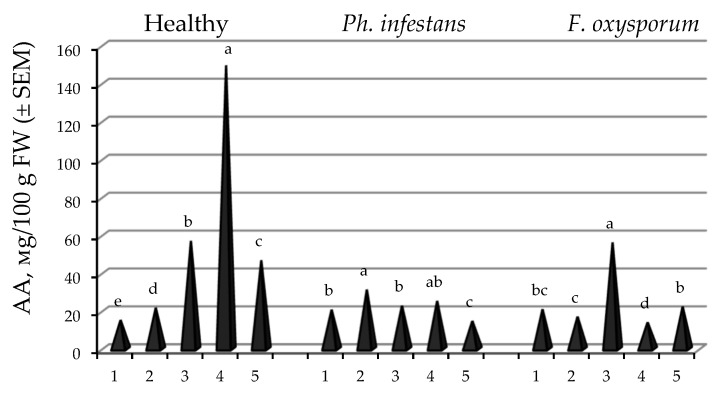
Effect of endophytic bacteria *B. subtilis* 10-4 and 26D both individually and in compositions with SA on AA content in healthy and infected by *Ph. infestans* and *F. oxysporum* stored potato tubers (time of storage 5 months). **1**—control (non-inoculated with *B. subtilis*), **2**—*B. subtilis* 10-4; **3**—*B. subtilis* 10-4 + SA; **4**—*B. subtilis* 26D; **5**—*B. subtilis* 26D + SA. The bars are the means of three repetitions ± SEM. Different letters indicate a significant difference between the means at the probability level of *p*  <  0.05.

It was discovered that bacterization of potato tubers before storage with endophytic bacteria *B. subtilis* 10-4 and 26D led to a significant increase in AA concentration up to 42–373% by the sixth month of storage (Figure 8). At the same time, use of *B. subtilis* 10-4 and *B. subtilis* 26D in combinations with SA resulted in additional increases in AA content, reaching 149.6 mg/100 g FW (for strain 10-4 + SA) and 46.9 mg/100 g FW (for strain 26D + SA). Therefore, the inclusion of SA in the composition with the strains in one case (strain 10-4) increases the accumulation of AA, and in the other (strain 26D), on the contrary, it even leads to a decrease accumulation in comparison with the individual use of strain 26D, although in comparison with the non-treated control, as before, it was significantly higher (by 306%). Infection by pathogenic fungi *Ph. infestans* and *F. oxysporum* also led to an increase in AA content by 56% and 36%, respectively. Simultaneously, treatment of infected tubers with strains 10-4 and 26D in practically all variants (except 26D + SA + *Ph. infestans* and 26D + *F. oxysporum*, where the indicators were at the level of non-treated control tubers) led to a more significant accumulation of AA (up to 204%). So, in *Ph. infestans*-infected conditions the treatment with *B. subtilis* 10-4 separately and in combination with SA increased the AA content by 204% and 149%, respectively. Under *F. oxysporum* infection, the increase was 130% (for strain 10-4), 620% (for strain 10-4 + SA) and 146% (for strain 26D + SA).

Thus, treatment with endophytic *B. subtilis* 10-4 and 26D both individually and in compositions with SA contribute to increasing AA concentrations both in healthy and pathogen (*Ph. infestans, F. oxysporum*)-infected stored potato tubers. In variants with the combined use of *B. subtilis* with SA, a more enhanced AA level was also observed (especially for 10-4 + SA) (Figure 8). It is probably that an increased content of AA, which has also been established as a preservative, is one of the causative factors for why potatoes look fresh in variants with the joint use of *B. subtilis* 10-4 and SA even after 5 months of storage and under conditions of infection. These findings are evidence in favor of AA participation in the launch of protective reactions and its important contribution to slowing the aging process of stored potato. This suggests that AA is also an important contributor in *Bacillus*-driven lengthening of the shelf-life and preservation of the appearance and biological value of stored potatoes.

## 3. Materials and Methods

### 3.1. Plant Material, Scheme of Experiments, and Storage Conditions

**Scheme 1:**
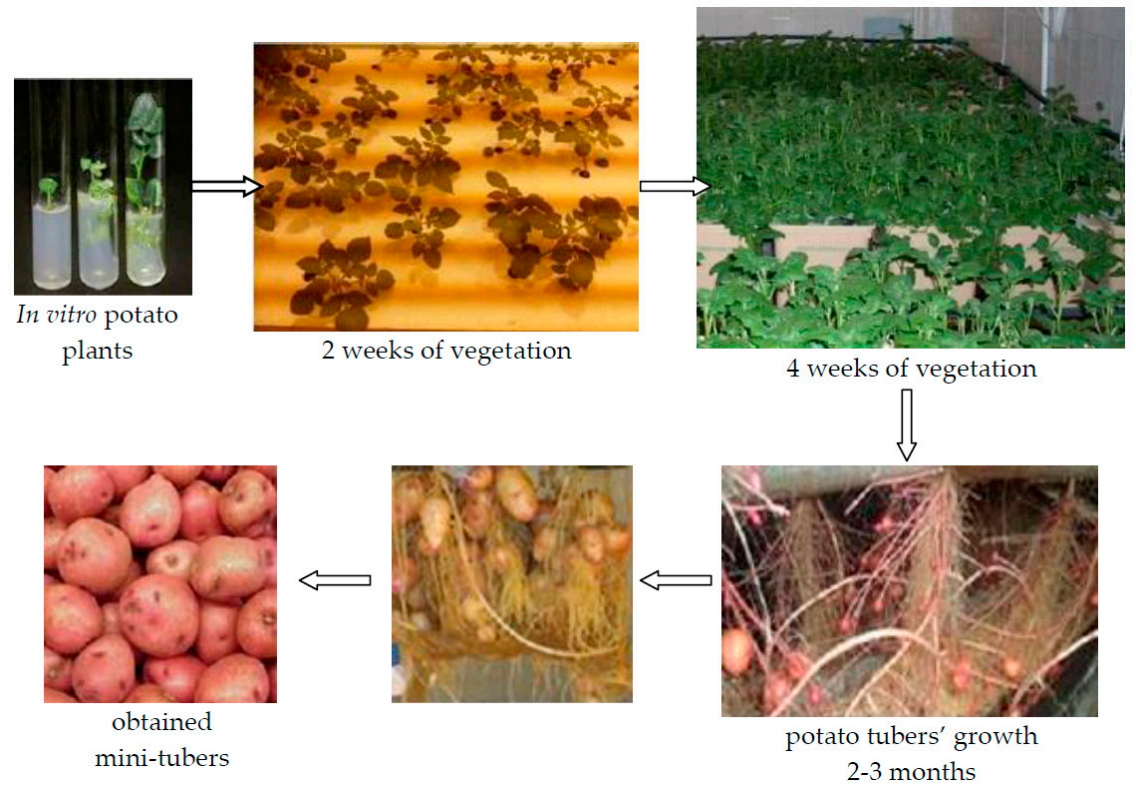
The scheme of producing hydroponically grown potato (*Solanum tuberosum* L., Cv. Bashkirsky) mini-tubers.

The experiments were carried out on virus-free potato (*Solanum tuberosum* L., Cv. Bashkirsky) mini-tubers grown using hydroponic equipment (“Minivit”, KD-10, Russia) at the laboratory of Potato Breeding and Seed Production at the Bashkir Research Institute of Agriculture UFRC RAS (Ufa, Russia) (Scheme 1). In vitro plants of healthy potatoes were placed in a hydroponic equipment and fixed with a support system, the roots were immersed in a continuously supplied nutrient solution of a commercial complex water-soluble fertilizer with microelements Novalon (19-19-19 + 2 MgO+ME, Novalon, DoctorTarsa, Turkey) (pH = 5.6 mol L) with a concentration of 0.4–0.6% (for one week), 0.8% (for two weeks), 1.2–1.4% (for three weeks), 1.5–1.8% (from 4 weeks to the end of the growing season). The lighting mode was divided into three main periods and amounted to 120 thousand, 150 thousand, and 80 thousand lux/hours, respectively. Obtained (freshly harvested) hydroponic mini-tubers of potato were characterized by an oval-rounded shape with medium-depth eyes, smooth red skin, and white flesh. The biomass of hydroponically grown mini-tubers was 4–6 g. The growing season is about 60–70 days.

**Scheme 2:**
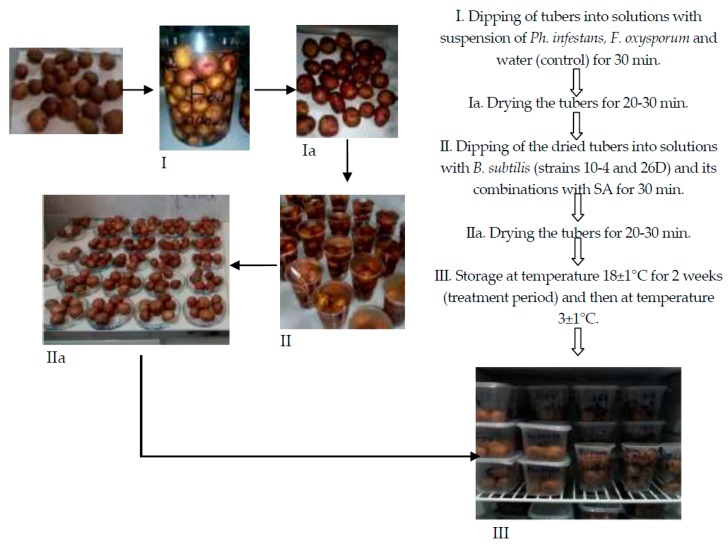
The scheme of hydroponically grown potato mini-tubers’ treatment with phytopathogenic fungi *Ph. infestans*, *F. oxysporum*, endophytic bacteria *B. subtilis* (strains 10-4 and 26D) and their combinations with SA before storage.

Immediately before storage, freshly harvested hydroponically grown potato mini-tubers were dipped into solutions with suspensions of phytopathogenic fungi *Ph. infestans* (Mont.) De Bary (10^8^ spores/mL), *F. oxysporum* (10^6^ spores/mL), and water (control) for 30 min and dried at 23 ± 1 °C (room temperature) for 20–30 min (Scheme 2). Then the dried tubers inoculated with a suspensions of *B. subtilis* (strains 10-4 and 26D) in concentrations 0, 10^3^, 10^4^, 10^5^, 10^6^, 10^7^, 10^8^ CFU/mL both individually and in compositions with SA (0.05 mM) [53] by immersing tubers into solutions for 30 min, then dried (20–30 min at 23 ± 1 °C) and stored (thermostat TVL-K 120, INSOVT, Russia) at a temperature of 18 ± 1 °C for 2 weeks and then at 3 ± 1 °C for 3–6 months (Scheme 2).

### 3.2. Bacterial Strains and Phytopathogenic Fungi

The endophytic bacterium *B. subtilis* strain 10-4 was previously isolated from arable soils of Republic of Bashkortostan (Russia) at the Bashkir Research Institute of Agriculture UFRC RAS (Ufa, Russia) and describe in detail [25] The strain *B. subtilis* 26D (the basis of the commercial biological product Phytosporin-M, BashInkom S&IE, Ltd., Ufa, Russia) was kindly provided by the microbiological laboratory of BashInkom S&IE, Ltd. (Ufa, Russia). *B. subtilis* (strains 10-4 and 26D) cells were cultured in potato glucose agar medium at 37 °C for 3–4 days [25,66]. To get inoculums of *B. subtilis* (strain 10-4 and 26D) with different cell concentrations we prepared the suspensions containing 10^8^ colony forming units (CFU)/mL of bacteria (strain 10-4 and 26D) according to 0.5 McFarland Turbidity Standard (additionally were monitored by the optical density at 600 nm (OD600) (SmartSpec^TM^ Plus spectrophotometer, Bio-Rad, Hercules, CA, USA)), and then diluted down to 10^7^, 10^6^, 10^5^, 10^4^, and 10^3^ CFU/mL using distilled water or/and solution of 0.05 mM salicylic acid (SA) in distilled water.

*Phytophthora infestans* (Mont.) de Bary (causative agent of potato late blight) and *Fusarium oxysporum* (causative agent of fusarium wilt and dry rot) were obtained from the collection of microorganisms of the laboratory of Plant-Microbe Interactions of the Bashkir Research Institute of Agriculture UFRC RAS (Ufa, Russia) and microbiological laboratory of BashInkom S&IE, Ltd. (Ufa, Russia). Phytopathogenic fungi were grown on potato-glucose agar (PGA) (pH = 6.6) at a temperature of 28 °C [66]. The concentrations of phytopathogenic fungi *Ph. infestans* (10^8^ spores/mL) and *F. oxysporum* (10^6^ spores/mL) were prepared using a Goryaev chamber [66].

### 3.3. Assessment Visual Symptoms of Disease Development

Visual symptoms of disease development were evaluated on a 5 point scale (0 points—no symptoms, 1 point—damage from 1 to 25%, 2 points—from 26 to 50%, 3 points—from 51 to 75%, 4 points—more than 75%; 5 points—100% completely affected). An analysis of the intensity of the development of diseases on tuber slices was evaluated [67].

### 3.4. Determination of the Ability of B. subtilis to Colonize Internal Tissues of Potato Tubers

The ability of the *B. subtilis* 10-4 and 26D strains to colonize the internal tissues of potato mini-tubers was determined using surface-sterilized potato tubers 2 weeks and 3 months after inoculation by bacteria suspensions. Stored tubers were submerged in a 70% ethyl alcohol solution for 5 min. Then ethanol was drained, and the tubers were rinsed with sterile water (repeated 3 times), then the water was drained and the tubers were dried (about 10–15 min). After surface sterilization, the tubers were cut in half (under sterile conditions) and the cut parts was laid out in Petri dishes with Luria-Bertani (LB) medium (1% bacto-tryptone, 0.5% yeast extract, 1% sodium chloride, 2% agar) and kept for 1 h in an thermostat at 26 °C, then the tubers were removed and the Petri dishes were left in the thermostat for 12 h at 26 °C for bacterial growth. Colony growth was recorded using photo documentation on the next day.

The identity of bacteria isolated from the internal tissues of tubers inoculated with *B. subtilis* 10-4 and 26D strains was determined using RAPD-PCR analysis [25].

Bacterial DNA was isolated using lysis buffer (1% tryptone×100, 1% tween-20, 1% Chelex 100 (Bio-Rad, Hercules, CA, USA), 0.005% cresol red, water) [25]. Genetic polymorphism of the strains was evaluated according to the results of RAPD-PCR of total DNA using AFK primers (5′-GCGTCCATTC-3′). Amplification was performed on Tercik equipment (DNA-Technology, Moscow, Russia). Analysis and visualization of products obtained as a result of RAPD analysis was performed using horizontal electrophoresis in 1.5% agarose gel in a SE-2 chamber (Helikon, Moscow, Russia) at 25 kV for 1 h. The gel was stained with ethidium bromide, and results recorded using the gel documentation system Gel Doc XR (Bio-Rad, Hercules, CA, USA).

### 3.5. Assessment the Quantitative Content (Titer) of B. subtilis Bacteria in Potato Tubers

The quantitative content (titer) of *B. subtilis* in potato tubers was determined by the classical method of titration [66].

### 3.6. Quantification of Proline (Pro) Content

Pro was determined according to Bates et al. [68]. 0.5 g fresh tissue of stored tubers was extracted with 2.5 mL boiled water. Then 2.0 mL extract was mixed with equal volume of acid ninhydrin solution (1.25 g ninhydrin dissolved in 30 mL glacial acetic acid and 20 mL of 6 M phosphoric acid) and glacial acetic acid. The samples were then incubated at 100 °C for 1 h and the reaction was terminated by cooling the tubes in an ice bath. After cooling, Pro was spectrophotometrically determined at 522 nm and the Pro content of each sample was obtained using a standard curve, based on mg/g FW.

### 3.7. Estimation of Lipid Peroxidation (MDA)

Lipid peroxidation was evaluated by measuring of the MDA concentration, the product of the lipid peroxidation reaction, according to the Health and Packer method [69]. Next, 0.2 g of fresh tubers were homogenized with 1 mL of 10% trichloroacetic acid and centrifuged at 10,000 rpm for 10 min. Then 1 mL of the supernatant was mixed with 20% trichloroacetic acid containing 0.25% thiobarbituric acid and was heated at 95 °C for 30 min, quickly cooled in an ice bath, and then centrifuged again at 10,000 rpm for 10 min. The absorbance of the supernatant was read at 532 and 600 nm. MDA concentration was calculated using an extinction coefficient of 155 mM^−1^cm^−1^ and was expressed as nmol g^−1^ FW.

### 3.8. Ascorbic Acid (AA) Determination

Ascorbic acid concentrations were estimated by titration method using 2,6-dichlorophenolindophenolate sodium [70]. One gram of fresh tubers were homogenized with 1 mL of hydrochloric acid and transferred to a volumetric flask and brought up to 100 mL with hydrochloric acid. Then the extract was kept for 10 min, mixed and filtered. 10 mL of obtained extract was titrated with a 2,6-dichlorophenolindophenolate sodium solution until a slightly pink color appeared without fading for 15–20 s. Total AA concentration was calculated by the formula specified in the methodology [70].

### 3.9. Statistical Analysis

All microbiological, molecular, biochemical and physiological experiments were performed at least in three biological and three analytical replicates. The data were presented as the mean ± standard error (SEM). Statistically significant differences between the mean values were evaluated two-way analysis of variance (ANOVA), followed by Tukey test (*p*  <  0.05).

## 4. Conclusions

In summary, the results obtained from this study and presented here established that the treatment of potato tubers immediately prior to storage with endophytic *B. subtilis* (10-4 and 26D) and its compositions with SA suppressed *Ph. infestans* and *F. oxysporum* development in a dose-dependent manner with the most effective concentrations established to be 10^8^ CFU/mL (10-4 and 26D), 10^7^ CFU/mL (10-4 + SA) and 10^6^ CFU/mL (26D + SA). Additional findings demonstrate the capacity of *B. subtilis* (10-4, 26D), both individually and in combination with SA, to penetrate/colonize the internal tubers’ supporting the conclusion that this phenomenon significantly promotes competition with pathogenic microorganisms in the early stages of pathogen damage allowing to compete effectively for nutrition substances and space with pathogenic fungi from the inside, in some cases almost completely suppressing their development. The obtained results showing reduced pathogen (*Ph. infestans* and *F. oxysporum*)-induced accumulation of Pro and MDA under the influence of *B. subtilis* (10-4, 26D) and *B. subtilis* (10-4, 26D) + SA and the ability of applied treatments increase AA concentrations support the conclusion that these cells are protected from the damaging effect of ROS and control of aging processes. Thus, our results revealed that endophytic *B. subtilis* (10-4, 26D) and their combinations with SA (especially *B. subtilis* 10-4 + SA) may be a useful technique to alleviate the *Ph. infestans* and *F. oxysporum* causing diseases development in potato tubers during storage with prolonging shelf-life and preserving fresh appearance/quality.
